# Functional evolution of the vitamin D and pregnane X receptors

**DOI:** 10.1186/1471-2148-7-222

**Published:** 2007-11-12

**Authors:** Erica J Reschly, Afonso Celso Dias Bainy, Jaco Joaquim Mattos, Lee R Hagey, Nathan Bahary, Sripal R Mada, Junhai Ou, Raman Venkataramanan, Matthew D Krasowski

**Affiliations:** 1 Department of Pathology, University of Pittsburgh, Pittsburgh, PA, USA; 2 Departamento de Bioquimica, CCB, Universidade Federal de Santa Catarina, Florianópolis, SC, Brazil; 3 Department of Medicine, University of California at San Diego, San Diego, CA, USA; 4 Department of Molecular Genetics and Biochemistry, University of Pittsburgh, Pittsburgh, PA, USA; 5 Department of Pharmaceutical Sciences, University of Pittsburgh, Pittsburgh, PA, USA

## Abstract

**Background:**

The vitamin D receptor (VDR) and pregnane X receptor (PXR) are nuclear hormone receptors of the NR1I subfamily that show contrasting patterns of cross-species variation. VDR and PXR are thought to have arisen from duplication of an ancestral gene, evident now as a single gene in the genome of the chordate invertebrate *Ciona intestinalis *(sea squirt). VDR genes have been detected in a wide range of vertebrates including jawless fish. To date, PXR genes have not been found in cartilaginous fish. In this study, the ligand selectivities of VDRs were compared in detail across a range of vertebrate species and compared with those of the *Ciona *VDR/PXR. In addition, several assays were used to search for evidence of PXR-mediated hepatic effects in three model non-mammalian species: sea lamprey (*Petromyzon marinus*), zebrafish (*Danio rerio*), and African clawed frog (*Xenopus laevis*).

**Results:**

Human, mouse, frog, zebrafish, and lamprey VDRs were found to have similar ligand selectivities for vitamin D derivatives. In contrast, using cultured primary hepatocytes, only zebrafish showed evidence of PXR-mediated induction of enzyme expression, with increases in testosterone 6β-hydroxylation activity (a measure of cytochrome P450 3A activity in other species) and flurbiprofen 4-hydroxylation activity (measure of cytochrome P450 2C activity) following exposure to known PXR activators. A separate assay in vivo using zebrafish demonstrated increased hepatic transcription of another PXR target, multidrug resistance gene (ABCB5), following injection of the major zebrafish bile salt, 5α-cyprinol 27-sulfate. The PXR target function, testosterone hydroxylation, was detected in frog and sea lamprey primary hepatocytes, but was not inducible in these two species by a wide range of PXR activators in other animals. Analysis of the sea lamprey draft genome also did not show evidence of a PXR gene.

**Conclusion:**

Our results show tight conservation of ligand selectivity of VDRs across vertebrate species from Agnatha to mammals. Using a functional approach, we demonstrate classic PXR-mediated effects in zebrafish, but not in sea lamprey or African clawed frog liver cells. Using a genomic approach, we failed to find evidence of a PXR gene in lamprey, suggesting that VDR may be the original NR1I gene.

## Background

The vitamin D receptor (VDR, NR1I1) and pregnane X receptor (PXR, NR1I2) are members of the nuclear hormone receptor (NR) superfamily of ligand-activated transcription factors. NRs work in concert with co-activators and co-repressors to regulate gene expression [1-3]. NRs share a modular domain structure, which includes, from N-terminus to C-terminus, a modulatory A/B domain, the DNA-binding domain (DBD; C domain), the hinge D domain, the ligand-binding domain (LBD; E domain) and a variable C-terminal F domain [3].

**Figure 1:**
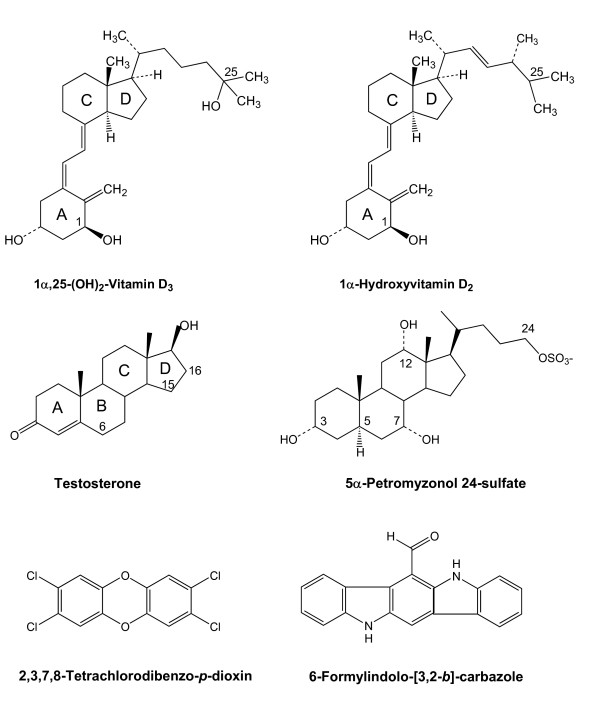
Chemical structures of 1α,25-(OH)_2_-vitamin D_3 _(calcitriol), 1α-hydroxyvitamin D_2_, testosterone, 5α-petromyzonol 24-sulfate (major sea lamprey bile salt), 2,3,7,8-tetrachlorodibenzo-*p*-dioxin (TCDD), and 6-formylindolo [3,2-*b*]carbazole. Select bond positions are numbered for the vitamins, testosterone, and 5α-petromyzonol 24-sulfate, and the lettering of the steroidal rings is indicated for calcitriol, 1α-hydroxyvitamin D_2_, and testosterone. The carbon atoms numbered for testosterone indicate the sites of hydroxylation in the species studied in this report.

VDRs bind 1α,25-(OH)_2_-vitamin D_3 _(calcitriol) with high affinity and mediate classic calcitriol effects such as regulation of calcium and phosphate homeostasis (see Figure 1 for chemical structure of calcitriol). Over the last two decades, VDRs have been shown to influence a variety of physiological functions, affecting nearly every organ and tissue [4-7]. VDR genes have been detected in mammals, birds, amphibians, reptiles, teleost fish, and sea lamprey (see Additional file 1 for sequence alignments) [8,9]. All mammalian genomes analyzed to date have a single VDR gene; where expression has been studied, VDR is expressed in a broad range of tissues that include brain, gut, heart, skeletal muscle, liver, pancreas, and immune tissues [9]. A similarly broad pattern of tissue expression is also seen with African clawed frog (*Xenopus laevis*) [10] and avian VDRs [11,12]. Some teleost fish, including pufferfish (*Takifugu rubripes*) and Japanese flounder (*Paralichthys olivaceus*) have two VDR genes [13,14]. Like mammalian, bird, and frog VDRs, the pufferfish VDRα and flounder VDRβ have widespread tissue distribution; in contrast, the pufferfish VDRβ is expressed only in gut while the flounder VDRα shows little or no expression in liver, gill, and skeletal muscle [13,14].

In contrast to VDRs, PXRs have broad ligand specificity and, at least in mammals and chicken, serve as a 'chemical defense' protein that senses toxic concentrations of a wide variety of endogenous and exogenous compounds and transcriptionally controls detoxification pathways in liver, intestine, and other organs [15,16]. PXR regulates the metabolism, transport, and excretion of bile salts, xenobiotics, steroid hormones, and vitamins. 'Classic' PXR transcriptional targets in mammals include the broad specificity cytochrome P450 (CYP) 2C and 3A enzymes, as well as transporters such as multidrug resistant protein (MDR, ABCB1) [17-22]. While the majority of PXR studies have been on mammalian species, studies of chicken PXR (also known as chicken X receptor, CXR) show similar transcriptional targets [23,24].

PXR genes have been cloned and functionally characterized from a variety of vertebrate species, including human, rhesus monkey, mouse, rat, rabbit, dog, pig, chicken, African clawed frog, and zebrafish [15,17,18,23,25-27]. The PXR LBD is unusually divergent across species compared to other NRs (see Additional file 1 for sequence alignments), and previous studies have shown significant differences in ligand specificity of PXRs across species [27-29]. Unlike VDR, PXR gene(s) have yet to be detected in cartilaginous fish.

In this study, we characterize in detail the ligand specificity of VDRs from three model non-mammalian species: sea lamprey (*Petromyzon marinus*; a jawless fish), zebrafish (*Danio rerio*, a teleost fish), and the African clawed frog (an amphibian). In addition, we study the single VDR/PXR-like NR from the chordate invertebrate *Ciona intestinalis *(sea squirt) [30], a member of Urochordata, a subphylum now thought to be the closest extant relatives of vertebrates [31]. We compare these VDRs to human and mouse VDRs (abbreviations: human VDR, hVDR; mouse VDR, mVDR; *Xenopus laevis *VDR, xlVDR; zebrafish VDR, zfVDR; sea lamprey VDR, lampVDR). We also probed the evolutionary origin of PXR and VDR in basal vertebrates. To this end, we searched for evidence of PXR-like gene(s) in sea lamprey both by comparative genomics and by functional assays in cultured primary hepatocytes. Primary hepatocyte culture systems were also developed and tested for zebrafish and African clawed frog. The zebrafish studies were carried further by studies of liver transcription following injection of zebrafish with bile salts. The results demonstrate that classic PXR effects, similar to those described in mammals and chicken, are evident in zebrafish liver and isolated hepatocytes. In contrast, there is no genomic or functional evidence of a PXR-like gene in sea lamprey.

## Results

### Ligand specificity of vertebrate VDRs

**Figure 2:**
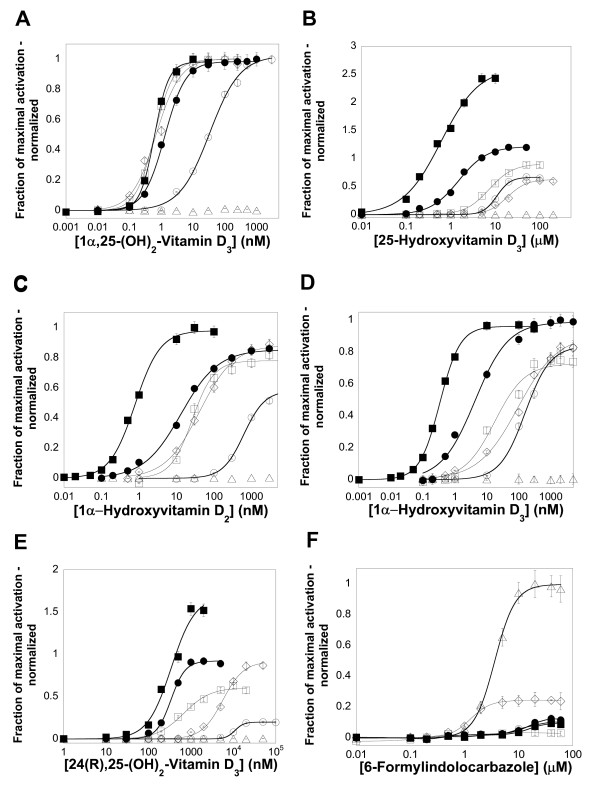
Concentration-response curves for activation of VDRs by vitamin D derivatives and 6-formylindolo [3,2-*b*]carbazole. The ordinate represents activation of VDR, relative to vehicle control, and normalized to the maximal activator (0.5 μM calcitriol for mouse and sea lamprey VDRs; 1 μM calcitriol for human, frog, and zebrafish VDRs; 20 μM 6-formylindolo [3,2-*b*]carbazole for *Ciona intestinalis *VDR/PXR). (**A**)-(**E**) Human (●), mouse (■), frog (○), zebrafish (□), and sea lamprey (◇) VDRs are all activated by vitamin D derivatives while the *Ciona intestinalis *VDR/PXR (Δ) is insensitive to all vitamin D compounds. (**F**) The planar molecule 6-formylindolo [3,2-*b*]carbazole activates most vertebrate VDRs (i.e., all except zebrafish VDR) weakly and the *Ciona *VDR/PXR at low micromolar concentrations.

To compare ligand specificity of mammalian and non-mammalian VDRs, luciferase-based reporter assays were used to study ligand activation of hVDR, mVDR, xlVDR, zfVDR, lampVDR, and *Ciona *VDR/PXR (ciVDR/PXR). All five vertebrate receptors were activated by 1α,25-(OH)_2_-vitamin D_3 _(calcitriol), 1α-hydroxyvitamin D_2_, 1α-hydroxyvitamin D_3_, 25-hydroxyvitamin D_3_, and 24(R),25-(OH)_2_-vitamin D_3 _(Figure 2A–E; Table 1). xlVDR has lower potency for the five vitamin D derivatives studied, similar to the initial report published on *X. laevis *VDR [10]. The other notable difference was that the two mammalian VDRs had higher apparent affinity for 1α-hydroxyvitamin D_2 _and 1α-hydroxyvitamin D_3 _than the three non-mammalian VDRs (Figure 2C and 2D). Otherwise, there were few major differences between the five receptors with regard to vitamin D derivatives. This is consistent with the high degree of sequence conservation across vertebrate VDRs at positions shown to interact with ligands in x-ray crystallographic structures of human [32-34], rat [35,36], and zebrafish VDRs [37,38] (Additional file 2). The ciVDR/PXR was not activated by any of the vitamin D derivatives (Figure 2A–E; Table 1).

**Table 1:** Effects of compounds on VDRs

**Common name**	**Significance**	**Human VDR** **EC_50 _± SD** **(relative efficacy)**	**Mouse VDR** **EC_50 _± SD** **(relative efficacy)**	***Xenopus *VDR** **EC_50 _± SD** **(relative efficacy)**	**Zebrafish VDR** **EC_50 _± SD** **(relative efficacy)**	**Sea lamprey VDR** **EC_50 _± SD** **(relative efficacy)**	***Ciona *VDR/PXR** **EC_50 _± SD** **(relative efficacy)**
1,25-(OH)_2_-Vitamin D_3_	Vitamin D	1.2 ± 0.2 nM (1.0^a^)	0.6 ± 0.1 nM (1.0^a^)	33 ± 3.5 nM (1.0^a^)	0.7 ± 0.1 nM (1.0^a^)	0.8 ± 0.1 nM (1.0^a^)	No effect
1α-Hydroxyvitamin D_2_	ligands	13 ± 1.2 nM (0.88)	0.8 ± 0.1 nM (0.98)	624 ± 49 nM (0.58)	26 ± 1.7 nM (0.78)	41 ± 3.6 nM (0.88)	No effect
1α-Hydroxyvitamin D_3_		4.3 ± 0.5 nM (0.99)	0.3 ± 0.04 nM (0.96)	157 ± 10 nM (0.84)	18 ± 1.4 nM (0.73)	94 ± 8.0 nM (0.90)	No effect
25-Hydroxyvitamin D_3_		1.5 ± 0.2 M (1.22)	0.6 ± 0.1 μM (2.62)	11 ± 0.8 nM (0.67)	7.3 ± 0.8 μM (0.91)	19 ± 2.1 μM (0.63)	No effect
24(R),25-(OH)_2_-Vitamin D_3_		0.3 ± 0.02 μM (0.9)	0.3 ± 0.02 μM (1.7)	11 ± 1.7 μM (0.20)	0.7 ± 0.06 μM (0.6)	5.6 ± 0.8 μM (0.9)	No effect
5α-Petromyzonol	Bile salts from	No effect	No effect	No effect	No effect	No effect	No effect
5α-Petromyzonol 24-sulfate	sea lamprey	No effect	No effect	No effect	No effect	No effect	No effect
5α-Cyprinol	Major bile salt	No effect	No effect	No effect	No effect	No effect	No effect
5α-Cyprinol 27-sulfate	for zebrafish and *Xenopus *species	No effect	No effect	No effect	No effect	No effect	No effect
Lithocholic acid	Major human	11 ± 0.7 (0.12)	7.1 ± 0.9 (0.36)	No effect	No effect	No effect	No effect
Glycolithocholic acid	secondary	No effect	16 ± 2.1 (0.28)	No effect	No effect	No effect	No effect
Taurolithocholic acid	bile acids and	No effect	26 ± 3.8 (0.23)	No effect	No effect	No effect	No effect
Lithocholic acid 3-sulfate	metabolities	No effect	No effect	No effect	No effect	No effect	No effect
3-Ketolithocholic acid		55 ± 8.1 (0.17)	20 ± 0.3 (0.58)	No effect	No effect	No effect	No effect
6-Formylindolo- [3,2-*b*]-carbazole	Miscellaneous	> 10 (~0.12)	> 10 (~0.08)	> 10 (~0.10)	~5 (< 0.03)	2.0 ± 0.3 (0.25)	0.86 ± 0.081 (1.0^a^)
2,3,7,8-Tetrachlorodibenzo-*p*-dioxin		No effect	No effect	No effect	No effect	No effect	0.23 ± 0.04 (1.80)

^a ^Maximal reference compound (efficacy = 1.0) ; efficacy of other compounds are relative to the maximal reference compound.

A 76-compound library of known nuclear hormone receptor ligands was screened for additional activators of xlVDR, zfVDR, and lampVDR (Additional file 3). None of these three VDRs were activated by farnesoid X receptor (NR1H4) or liver X receptor α (NR1H3) or β (NR1H2) ligands such as T-0901317, fexaramine, GW3965, or GW4064, or by steroid hormones such as 17β-estradiol or testosterone (Additional file 3). An unexpected finding was that 6-formylindolo- [3,2-*b*]-carbazole, a tryptophan photoproduct that is a high affinity agonist of the aryl hydrocarbon receptor [39], weakly activated the VDRs in the low micromolar range (Figure 2F; Table 1; see Figure 1 for chemical structure). This planar compound more strongly activated the ciVDR/PXR (Figure 2F; Table 1). The dioxin compound 2,3,7,8-tetrachlorodibenzo-*p*-dioxin (TCDD) also activated the ciVDR/PXR but not the vertebrate VDRs (Table 1). Like 6-formylindolo- [3,2-*b*]-carbazole, TCDD is also a planar compound (see Figure 1 for chemical structures).

### Studies of zebrafish primary hepatocytes

We next focused on studies of PXR in non-mammalian species. A major transcriptional target of PXR and VDR in mammals and birds is cytochrome P450 (CYP) 3A, a subfamily of enzymes with broad ligand specificity. A common functional assay for CYP3A activity is steroid (typically testosterone) 6β-hydroxylation – this activity is often used to measure CYP3A induction by xenobiotics or other compounds [40,41]. Exposure to PXR agonists increases testosterone 6β-hydroxylation in primary human and rodent hepatocytes [42,43], as well as in the chicken liver LMH cell line [24]. VDR agonists upregulate CYP3A more prominently in the intestine than liver in humans [44,45].

**Figure 3:**
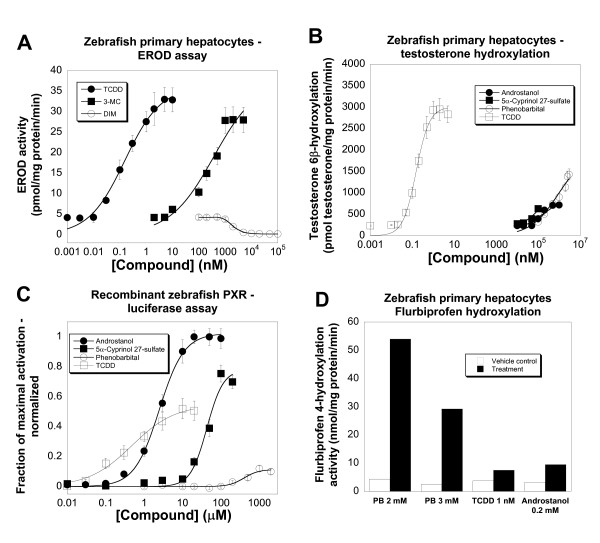
Enzyme induction in cultured primary zebrafish hepatocytes. (**A**) 2,3,7,8-Tetrachlorodibenzo-*p*-dioxin (TCDD) and 3-methylcholanthrene (3-MC) both increase EROD activity in zebrafish hepatocytes, relative to vehicle-only control, following a 48-hour exposure. In contrast, 3,3'-diindoylmethane (DIM) causes a reduction in EROD activity relative to vehicle-only control. (**B**) 5α-androstan-3α-ol (androstanol), 5α-cyprinol 27-sulfate, phenobarbital, and TCDD cause a concentration-dependent increase in testosterone 6β-hydroxylation activity in primary zebrafish hepatocytes, relative to vehicle control, following a 48-hour exposure. (**C**) Androstanol, 5α-cyprinol 27-sulfate, phenobarbital, and TCDD all activate recombinant zebrafish PXR using a luciferase-based reporter assay system in HepG2 liver cells. All values are normalized relative to 20 μM androstanol (which is assigned an efficacy of 1) and to β-galactosidase expression. (**D**) Phenobarbital (PB; 2 and 3 mM), TCDD (1 nM), and androstanol (0.2 mM) all increase flurbiprofen 4-hydroxylation activity in primary zebrafish hepatocytes, relative to vehicle control, following a 48-hour exposure.

Other than studies in the chicken LMH cell line mentioned above, little is known about enzyme induction in other non-mammalian species [46]. To this end, we developed a zebrafish primary hepatocyte cell culture model, adapting a protocol previously published by Collodi and colleagues [47]. As an initial test of enzyme induction, we first analyzed the ability of compounds to induce ethoxyresorufin *O*-deethylase (EROD) activity as a measure of CYP1A-like activity typical of the aryl hydrocarbon receptor pathway [48]. Similar to results previously reported for the zebrafish ZFL liver cell line [49], 24 hour exposure of the zebrafish cells to the dioxin compound TCDD strongly increased EROD activity (EC_50 _= 0.15 ± 0.022 nM, maximal rate = 33.0 pmol/min/mg protein; Figure 3A). 3-Methylcholanthrene (3-MC) also induced EROD activity with similar efficacy but lower potency than TCDD (EC_50 _= 373 ± 22 nM, maximal rate = 28.1 pmol/min/mg protein; Figure 3A). In contrast, 3,3'-diindoylmethane (DIM) at concentrations of 2 μM and greater caused a decrease in EROD activity (Figure 3A). A variety of other compounds were tested and found not to affect EROD activity in the zebrafish hepatocytes; these included calcitriol, 5α-androstan-3α-ol (androstanol), 5α-cyprinol (5α-cholestan-3α,7α,12α,26,27-pentol ) 27-sulfate (major bile salt of zebrafish [50]), nifedipine, and phenobarbital (data not shown).

Next, the ability of compounds to increase testosterone hydroxylation was tested in the zebrafish hepatocytes. Vehicle-treated zebrafish hepatocytes demonstrated basal 6β-, 15α-, 16α-, and 16β-hydroxylation activity as demonstrated by high-performance liquid chromatography (HPLC) analysis (data not shown; see Figure 1 for numbering of carbon skeleton of testosterone). In response to either androstanol or phenobarbital, 6β-hydroxylation was increased significantly (EC_50 _for androstanol = 20.0 ± 2.7 μM, maximal rate = 717 pmol/min/mg protein; EC_50 _for phenobarbital = 928 ± 109 μM, maximal rate = 1430 pmol/min/mg protein; Figure 3B). These two compounds have been previously described as zebrafish PXR agonists [27,29]. In contrast to 6β-hydroxylation, 15α-, 16α-, and 16β-hydroxylation activities were not influenced by incubation with androstanol, phenobarbital, or any other drugs tested (data not shown). Varying the exposure time of the inducers revealed that optimal induction was achieved with 48-hour exposure. Incubation with calcitriol (1–500 nM) did not increase 6β-hydroxylation of testosterone. Nifedipine (3–20 μM) and 5α-cyprinol 27-sulfate (20–200 μM) both produced small but reproducible increases in 6β-hydroxylation activity compared to vehicle-treated levels (approximately 15–20% increase compared to vehicle-treated control; Figure 3B). Somewhat unexpectedly, TCDD was found to be a potent and efficacious inducer of testosterone 6β-hydroxylation (EC_50 _= 0.154 ± 0.03 nM, maximal rate = 2830 pmol/min/mg protein; Figure 3B).

The ability of androstanol, 5α-cyprinol 27-sulfate, and phenobarbital to increase 6β-hydroxylation of testosterone in the zebrafish hepatocytes occurred at similar concentrations to those that activated recombinant zebrafish PXR, as measured by a luciferase-based assay in HepG2 cells (Figure 3C). The efficacy of these three compounds for increasing testosterone 6β-hydroxylation were, however, different; for example, phenobarbital has lower efficacy than androstanol for activating recombinant zebrafish PXR (Figure 3C) but higher efficacy in increasing testosterone 6β-hydroxylase activity in primary zebrafish hepatocytes (Figure 3B). These discrepancies may result in part from metabolism of steroid hormones and/or bile salts by the hepatocytes as opposed to HepG2 cells. TCDD was also found to activate zebrafish PXR (Figure 3C) but at much higher concentrations than needed to induce testosterone 6β-hydroxylation (Figure 3B). In fact, the concentrations of TCDD that increased testosterone 6β-hydroxylation are very close to those that increased EROD activity in the zebrafish hepatocytes (Figure 3A), suggesting that a common mechanism (e.g., aryl hydrocarbon receptor) mediates both effects.

A limited number of compounds were also tested for the ability to hydroxylate flurbiprofen, a measure of CYP2C9 activity in humans [51,52]. Zebrafish hepatocytes had basal flurbiprofen 4-hydroxylation activities of approximately 4 nmol/min/mg protein. This activity was markedly higher in hepatocytes exposed to phenobarbital (Figure 3D), with maximal activities greater than 50 nmol/min/mg protein in phenobarbital-treated cells (Figure 3D). Lesser increases relative to vehicle control were seen with treatments by TCDD (1 nM) or androstanol (200 μM; Figure 3D).

### Effects of bile salts on *in vivo *transcription in zebrafish livers

To further probe the effects of endogenous PXR activators on zebrafish liver *in vivo*, zebrafish were separately injected with each of four bile salts or vehicle controls. These included 5α-cyprinol 27-sulfate (major excreted bile salt of zebrafish [50] and an efficacious activator of recombinant zebrafish PXR [29]), 5β-scymnol (5β-cholestan-3α,7α,12α,24,26,27-hexol) 27-sulfate (weaker activator of recombinant zebrafish PXR [29,53]), as well as unconjugated 5α-cyprinol and 5β-scymnol (both inactive at recombinant zebrafish PXR [29,53]). Zebrafish were then sacrificed and liver mRNA isolated. As measured by semi-quantitative reverse transcription (RT)-PCR, none of the four bile salts produced any significant effect on transcription of β-actin (vehicle control – 60.0 ± 26.2 arbitrary units; 5α-cyprinol – 44.7 ± 19.6; 5α-cyprinol 27-sulfate – 38.2 ± 20.7; 5β-scymnol – 62.2 ± 21.8; 5β-scymnol 27-sulfate – 39.3 ± 17.5) or glyceraldehyde 3-phosphate dehydrogenase (GAPDH; vehicle control – 1.63 ± 0.54 arbitrary units normalized to β-actin; 5α-cyprinol – 1.50 ± 0.88; 5α-cyprinol 27-sulfate – 2.38 ± 0.79; 5β-scymnol – 1.17 ± 0.58; 5β-scymnol 27-sulfate – 1.08 ± 0.50). Relative to vehicle control, 5α-cyprinol 27-sulfate, but not the other three bile salts, produced a significant increase in transcription of MDR1 (tentatively classified as ABCB5 in zebrafish) as measured by semi-quantitative reverse transcription RT-PCR (vehicle control – 1.25 ± 0.25 arbitrary units normalized to β-actin; 5α-cyprinol – 2.00 ± 1.00; 5β-cyprinol 27-sulfate – 2.29 ± 0.75; 5β-scymnol – 1.08 ± 0.17; 5β-scymnol 27-sulfate – 1.50 ± 0.58; effect by 5α-cyprinol 27-sulfate significant at *p *< 0.01 relative to vehicle control). The bile salts tested did not produce significant effects on transcription of PXR (vehicle control – 1.04 ± 0.54 arbitrary units normalized to β-actin; 5α-cyprinol – 0.58 ± 0.33; 5α-cyprinol 27-sulfate – 1.50 ± 0.67; 5β-scymnol – 0.92 ± 0.38; 5β-scymnol 27-sulfate 0.75 ± 0.33) or CYP3C1 (vehicle control – 1.17 ± 0.33 arbitrary units normalized to β-actin; 5α-cyprinol – 1.33 ± 0.33; 5α-cyprinol 27-sulfate – 1.71 ± 0.50; 5β-scymnol – 1.17 ± 0.50; 5β-scymnol 27-sulfate 1.33 ± 0.58). These results are limited by possible metabolism of the bile salts following injection in zebrafish but do confirm that the major bile salt of zebrafish can produce classic PXR effects in zebrafish liver, including increased transcription of MDR, an effect that can be mediated by PXR in mammals [19,54].

### Studies of African clawed frog primary hepatocytes

Adapting a previously published protocol [55], we also cultured primary hepatocytes from adult African clawed frogs (*Xenopus laevis*). TCDD markedly increased EROD activity following a 48 hour exposure although with lower potency and efficacy than in the zebrafish hepatocytes (EC_50 _= 6.5 ± 0.77 nM, maximal rate = 9.8 pmol/min/mg protein). These results are consistent with previous reports showing lower sensitivity of *Xenopus laevis *aryl hydrocarbon receptors to TCDD [56,57]. The frog hepatocytes showed significant 6β- and 12α-hydroxylation of testosterone ; however, neither activity was substantially increased relative to vehicle control by treatment with dexamethasone, calcitriol, 5α-cyprinol, 5α-cyprinol 27-sulfate (major *Xenopus laevis *bile salt; Hagey LR, unpublished data), TCDD, or the *Xenopus laevis *PXRα endogenous agonist 3-aminoethylbenzoate [25,58] (Additional file 4). Thus, unlike zebrafish, testosterone hydroxylation by frog hepatocytes is not induced by activators of frog PXRs (also known as benzoate X receptors) or aryl hydrocarbon receptors.

### Studies of sea lamprey primary hepatocytes

Sea lampreys are a member of the jawless fish (Agnatha), a paraphyletic superclass of the phylogenetically most basal vertebrates. Using an adaptation of previously published methods for culturing primary sea lamprey tissues [59], we developed a primary cell culture method for sea lamprey hepatocytes. Initial experiments utilizing larval stage lampreys (i.e., prior to the transformer life-stage capable of parasitic feeding on live fish) were unsuccessful due to frequent contamination with bacteria and fungi, despite trying multiple antibiotic combinations and different decontamination techniques. Similar problems were also mentioned by Ma and Collodi [59]. Culture of hepatocytes from transformer stage lampreys were more successful and yielded cells that grew for at least 7–10 days. We were unable to passage the cells.

In contrast to the zebrafish hepatocytes, the sea lamprey hepatocytes demonstrated undetectable EROD activity (< 1 pmol/min/mg protein), both at baseline and after 24–48 hr exposure to various compounds. The following compounds did not alter EROD activity: TCDD, 5α-petromyzonol (5α-cholan-3α,7α,12α,24-tetrol), 5α-petromyzonol 24-sulfate (major sea lamprey bile salt [60]; see Figure 1 for chemical structure), androstanol, calcitriol, 3-MC, and phenobarbital. These results are consistent with that of a previous study that showed low basal EROD activity in microsomes from sea lamprey livers and, additionally, no inducibility of EROD activity in sea lampreys treated with compounds that act as efficacious aryl hydrocarybon receptor agonists in teleosts and terrestrial vertebrates [61].

The sea lamprey hepatocytes showed significant testosterone 6β-hydroxylation activity with an average basal activity of 458 pmol/min/mg protein. No other hydroxylated metabolites of testosterone were detected by HPLC even after incubating the hepatocytes with 250 μM testosterone for up to 24 hours. A wide range of compounds were tested for the ability to induce testosterone hydroxylation in lamprey hepatocytes including androstanol (1–200 μM), 5α-androst-16-en-3α-ol (1–200 μM), calcitriol (10–1000 nM), dexamethasone (0.1–50 μM), 5α-petromyzonol (0.01–20 μM; major unconjugated bile salt of sea lampreys [60]), 5α-petromyzonol 24-sulfate (0.1–100 μM; major excreted bile salt of sea lampreys [60]), 3-keto-5α-petromyzonol (0.01–20 μM), 3-MC (0.1–10 μM), phenobarbital (10–3000 μM), pregnenolone (1–200 μM), pregnenolone sulfate (10–200 μM), TCDD (1–10 nM), and 25-hydroxyvitamin D_3 _(0.1–5 μM). None of these compounds produced significant increases in testosterone 6β-hydroxylation. The lamprey hepatocytes also were able to 4-hydroxylate flurbiprofen (basal activity 2.8 nmol/min/mg protein). Flurbiprofen 4-hydroxylation was significantly decreased by 48 hour treatment with 5α-petromyzonol (20 μM; 0.21 nmol/min/mg protein) and 3-MC (5 μM; 0.33 nmol/min/mg protein).

Finally, we also looked for evidence of PXR gene(s) in the preliminary assembly of the sea lamprey genome (5.9X assembly, 21 Feburary 2007, Genome Sequencing Center [62], Washington University, Saint Louis, MO, USA). We searched for potential PXR ortholog(s) in sea lamprey by a reciprocal BLAST analysis strategy [63]. BLAST queries using DBD, LBD, and full-length protein sequences of all available vertebrate PXRs and CARs (constitutive androstane receptors; NR1I3; a receptor closely to PXRs) against translated nucleotides of the sea lamprey draft genome (tblastn) did not reveal any gene fragments that, when BLASTed against the Genbank nr database [64], reciprocally returned PXR or CAR genes. These analyses did, however, detect the already described sea lamprey VDR [8] and a novel, putative sea lamprey ortholog to farnesoid X receptor (FXR; NR1H4). Several contigs corresponded to the published cDNA sequence for the sea lamprey VDR Genbank: AY249863[8]: these included contig 990 (nucleotides 3761–3877, 6462–6563, 8284–8362, 13878–14009), contig 16240 (nucleotides 3538–3621), contig 20222 (nucleotides 13373–13570), and contig 21479 (nucleotides 13047–13304, 17009–17143, 17905–18072). The contigs that likely correspond to a lamprey FXR are contig 5004 (nucleotides 25341–25445, 27183–27422), contig 40984 (nucleotides 16–165), and contig 56284 (nucleotides 3555–3698). The lamprey FXR-like gene fragments showed closest sequence identity to the recently cloned and characterized FXR for the little skate (*Leucoraja erinacea*, a cartilaginous fish; Genbank: EF520727) [65]. Similar to CARs and PXRs, no putative orthologs to liver X receptors (LXRs; NR1H2 and NR1H3) were detected as well. In summary, although more complete sequencing of the sea lamprey genome may reveal additional NR1H and 1I genes, the evidence so far suggests the presence of FXR and VDR only, fewer than the inventory found in teleost fish (generally LXR, FXR, one or two VDRs, PXR), African clawed frog (LXR, FXR, VDR, and two PXRs), chicken (LXR, FXR, VDR, PXR) or mammals (LXRα, LXRβ, FXR, VDR, PXR, CAR).

## Discussion

**Figure 4:**
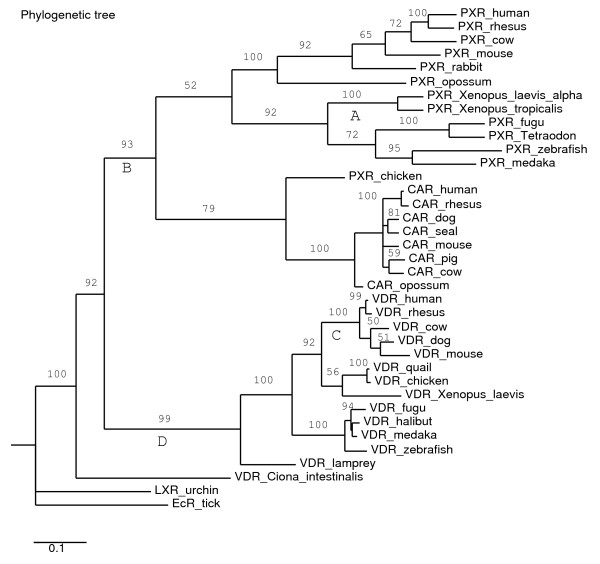
Phylogeny of vitamin D receptors (VDRs, NR1I), pregnane X receptors (PXRs,NR1I2), and constitutive androstane receptors (CARs, NR1I3). See Methods for details on the phylogenetic analysis. Four key evolutionary transitions in ligand sensitivity (i.e., ability to be activated by ligands) are proposed and indicated by the labels A, B, C, and D underneath four branches. Label A indicates loss of ligand sensitivity in the *Xenopus *frog PXRs relative to other PXRs. The *Xenopus laevis *and *Xenopus tropicalis *PXRs have narrow ligand sensitivity (essentially to benzoate compounds only) compared to other vertebrate PXRs. Label B indicates broadening of ligand specificity relative to the ancestral receptor. This is supported by the broader ligand specificity of vertebrate PXRs relative to the *Ciona intestinalis *VDR/PXR. Label C indicates acquisition of sensitivity to certain bile acids, particularly lithocholic acid and its derivatives, in mammalian VDRs. All non-mammalian VDRs studied so far are insensitive to bile salts. Label D indicates acquisition of sensitivity to vitamin D, a property of all vertebrate VDRs but not the *Ciona *VDR/PXR.

VDR and PXR are closely related NRs in the NR1I subfamily that we previously proposed to have arisen from a duplication of an ancestral gene [9]. Although at first glance these two NRs appear to be quite different, they bind to similar response elements in the promoter of target genes and also share a number of target genes including CYP3A enzymes [17,19-22,25,44,66,67]. Some of the key evolutionary transitions in ligand selectivity for NR1I receptors are indicated in the phylogeny shown in Figure 4. In this report, we show that VDRs have similar selectivity for vitamin D ligands across a range of vertebrate species that include sea lamprey (Agnatha, jawless fish), zebrafish (teleost fish), African clawed frog (amphibian), mouse, and human (mammals). Although the functions of vitamin D in basal vertebrates are not well understood, the ability to bind and be activated by vitamin D ligands has been conserved across vertebrates.

PXRs, on the other hand, show extensive sequence divergence across species [27,29,53]. In this report, we demonstrate that PXR activators can produce effects similar to those described in mammals and chicken in both the primary hepatocytes and livers of zebrafish. These effects include increases in CYP3A-like activity (testosterone 6β-hydroxylation), CYP2C-like activity (flurbiprofen 4-hydroxylation), and mRNA transcription of MDR1. In zebrafish, both synthetic (e.g., phenobarbital) and endogenous (e.g., 5α-cyprinol 27-sulfate) ligands can produce these effects. In contrast, classic PXR-like effects were not elicited in either sea lamprey or African clawed frog primary hepatocytes by a range of compounds that activate PXRs from other species. The PXRs for the African clawed frog are very divergent from other species in multiple respects: (1) ligand selectivity (narrow selectivity for benzoates, no xenobiotic sensitivity), (2) tissue expression pattern (little expression by liver or intestine, high expression in brain, ovary, skin, and testis), and (3) developmental expression pattern (strong expression during tadpole development, less expression in adults) [25,27,58,68,69]. Our results provide further evidence that additional xenobiotic-response PXRs or related NRs are not found in the African clawed frog genome. It is an unresolved evolutionary question why African clawed frog (and perhaps other amphibians) lack an NR such as CAR or PXR that can respond to a diverse assortment of potentially toxic endogenous and exogenous molecules [58].

The NR repertoire in jawless fish has not been well-studied, with the exception of VDR [8] and sex and adrenocortical steroid receptors [70,71]. As one of the most basal extant vertebrates, these animals can provide helpful evolutionary perspective. For the sea lamprey, we were unable to find any compounds that increased testosterone 6β-hydroxyation or fluribiprofen 4-hydroxylation in primary hepatocytes (both possible consequences of PXR-mediated transcriptional regulation), even though the lamprey hepatocytes had substantial basal levels of both activities. Coupled with our failure to find a PXR-like gene in the preliminary release of the sea lamprey genome (despite finding multiple contigs that contained fragments of a putative FXR gene in addition to the already characterized VDR gene), the available data suggest that PXR gene(s) may not exist in the sea lamprey. If true, then VDR is either the evolutionarily older NR1I or, alternatively, PXR gene(s) have been lost during some or all jawless fish lineages.

The properties of the putative *Ciona intestinalis *ortholog to VDR and PXR demonstrate that invertebrate and vertebrate NR1I receptors have diverged markedly in ligand selectivity. The ciVDR/PXR does not respond to vitamin D ligands and, in a separate manuscript, we will report lack of sensitivity of the ciVDR/PXR to bile salts, steroids, typical PXR-activating xenobiotics, and fat-soluble vitamins other than vitamin D (i.e., vitamins A, E, and K). The ciVDR/PXR was found to be activated only by a small number of planar, synthetic compounds including *n*-butyl-*p*-aminobenzoate, carbamazepine, 6-formylindolo- 3,2-*b*-carbazole, and TCDD (EJ Reschly, MD Krasowski, unpublished data). There are no clear correlates of vitamin D or bile salts yet described in invertebrates, and as a result, endogenous ligands for the ciVDR/PXR would likely be different from those for vertebrate VDRs and PXRs. *Ciona intestinalis *is, however, capable of synthesizing steroid hormones and also accumulates cholesterol and other sterols from dietary sources [72,73]. The endogenous activators of the ciVDR/PXR may be as yet undescribed steroidal molecules that have structural similarity to vertebrate vitamins and/or bile salts. Alternatively, ciVDR/PXR may be activated by exogenous ligands relevant to its marine environment.

## Conclusion

Our results show that VDR ligand selectivity is highly conserved across vertebrate species. Using primary hepatocyte and *in vivo *models, we demonstrate classic PXR-mediated effects in liver cells from zebrafish but not the African clawed frog. Using functional and comparative genomic approaches, we failed to find evidence of PXR gene(s) in sea lamprey, suggesting VDR may be the evolutionarily older gene or that PXR gene(s) have been lost in some cartilaginous fish lineages. Vertebrate VDRs and PXRs have markedly different ligand selectivity from the VDR/PXR from the chordate invertebrate *Ciona intestinalis *indicating substantial functional divergence during evolution.

## Methods

### Animals

Adult *Xenopus laevis *frogs were purchased from NASCO (Fort Atkinson, WI, USA). The AB strain was used for the zebrafish experiments. Juvenile and transformer stage sea lampreys were obtained from Acme Lamprey Company (Harrison, ME, USA). All animal studies were performed in conformity with the Public Health Service Policy on Human Care and Use of Laboratory Animals, incorporated in the Institute for Laboratory Animal Research Guide for Care and Use of Laboratory Animals. Vertebrate animal studies were approved by the University of Pittsburgh Institutional Animal Care and Use Committee (hepatocyte studies in African clawed frog, zebrafish, and sea lamprey), the Committee on Animal Use of the University of California at San Diego (bile isolation and purification studies), and Ethics Committee of the Federal University of Santa Catarina (*in vivo *zebrafish liver studies).

### Chemicals

The sources of the chemicals were as follows: *n*-butyl-*p*-aminobenzoate, ethyl 3-aminobenzoate, 1,25-(OH)_2_-vitamin D_3_, glycocholic acid, taurocholic acid, fexaramine, GW3965, GW4064, phenobarbital (Sigma, St. Louis, MO, USA); 5α-cholic acid, 5α-petromyzonol, 5α-petromyzonol 24-sulfate, 3-ketopetromyzonol sulfate (Toronto Research Chemical, Inc., North York, ON, Canada); TCDD (Cambridge Isotopes, Andover, MA); 1α-hydroxyvitamin D_2_, 1α-hydroxyvitamin D_3 _(EMD Chemicals, San Diego, CA, USA); 24(R),25-(OH)_2_-vitamin D_3_, 25-hydroxyvitamin D_3_, Nuclear Receptor Ligand Library (76 compounds known as ligands of various nuclear hormone receptors; BIOMOL International, Plymouth Meeting, PA, USA). 5α-cyprinol 27-sulfate was isolated from Asiatic carp (*Cyprinus carpio*) bile [74]. 5β-scymnol 27-sulfate was isolated from the bile of Spotted eagle ray (*Aetobatus narinari*). Bile salts were purified by extraction and Flash column chromatography. Bile alcohol sulfates were chemically deconjugated (e.g., to 5α-cyprinol and 5β-scymnol) using a solution of 2,2-dimethoxypropane:1.0 *N *HCl, 7:1 v/v, and incubating 2 hours at 37°C, followed by the addition of water and extraction into ether. Completeness of deconjugation and assessment of purity was performed by thin-layer chromatography using known standards. Other than those described above, steroids and bile salts were obtained from Steraloids (Newport, RI, USA).

### Maintenance of cell lines

The creation of a HepG2 (human liver) cell line stably expressing the human Na^+^-taurocholate cotransporter (NTCP; SLC10A1) has been previously reported [29,53]. HepG2-NTCP cells were grown in modified Eagle's medium-α containing 10% fetal bovine serum and 1% penicillin/streptomycin. The cells were grown at 37°C in 5% CO_2_. The zebrafish ZFL liver cell line (ATCC) was grown in 50% Leibovitz's L-15 medium with 2 mM *L*-glutamine, 35% Dulbecco's modified Eagle's medium with 4.5 g/L glucose and 4 mM *L*-glutamine, 15% Ham's F-12 with 1 mM *L*-glutamine supplemented with 0.15 g/L sodium bicarbonate, 15 mM HEPES, 10 μg/mL human insulin (Sigma), 50 ng/mL recombinant human epidermal growth factor (Sigma), and 5% fetal bovine serum. ZFL cells were grown at 28°C in room air. The *Xenopus laevis *A6 kidney cell line (ATCC, Manassus, VA, USA) was grown in 75% NCTC 109 medium, 15% distilled water, and 10% fetal bovine serum at 26°C in 2% CO_2_. Except as noted above, all media and media supplements for the HepG2, ZFL, and A6 cell lines were obtained from Invitrogen (Carlsbad, CA, USA).

### Molecular biology

Plasmids containing human VDR, zebrafish PXR, human organic anion transporting polypeptide (SLC21), as well as the reporter constructs tk-UAS-Luc and CYP3A4-PXRE-Luc, and 'empty' vectors pCDNA, PsG5, and PM2 were generously provided by SA Kliewer, JT Moore, and LB Moore (GlaxoSmithKline, Research Triangle Park, NC, USA). Mouse VDR (IMAGE clone 3710866) and pCMV-sport6 vectors were obtained from Invitrogen (Carlsbad, CA). The expression vectors were either full-length receptors (i.e., containing both a DBD and LBD; hVDR and mVDR) or GAL4/VDR chimeras that contain only the LBD of the VDR (xlVDR, zfVDR, lampVDR, ciVDR/PXR). For the full-length expression vectors, the reporter plasmid was CYP3A4-PXRE-Luc, a construct that contains a promoter element from CYP3A4 (recognized by VDR DBDs) driving luciferase expression. For the GAL4/LBD expression constructs, the reporter plasmid was tk-UAS-Luc, which contains GAL4 DNA binding elements driving luciferase expression. xlVDR was cloned by RT-PCR from total RNA extracted from the frog A6 cell line. zfVDR was cloned by RT-PCR from total RNA extracted from the ZFL liver cell line. lampVDR was cloned by PCR from a full-length sea lamprey VDR clone, described as the 'insertless' full-length cDNA [8], generously provided by G.K. Whitfield (University of Arizona College of Medicine, Tucson, AZ, USA). The LBDs of xlVDR (amino acid residues 90–422) [Genbank: U91846], zfVDR (amino acid residues 121–453) [Genbank: AF164512], and lampVDR (amino acid residues 92–406) [Genbank: AY249863] were inserted into the pM2-GAL4 vector to create GAL4/LBD chimeras. Details of the cloning of the ciVDR/PXR are being described in a separate report. The ciVDR/PXR LBD construct contains amino acid residues 57–391 [Genbank: BR000137].

### Co-transfections and transactivation assays

The basic methodology for the luciferase reporter assays in 96-well format was as follows. On day 1, cells were seeded onto 96-well white opaque plates at 30,000 cells/well. On day 2, the medium was exchanged, and cells were transfected using calcium phosphate precipitation. Ligand activation of VDRs was determined by a luciferase-based functional assay using the HepG2-NTCP cells as previously described [29]. For hVDR, 3.5 ng/well of VDR plasmid was co-transfected with 30 ng/well of the reporter CYP3A4-PXRE-Luc and 20 ng/well of pSV-μ-galactosidase (Promega, Madison, WI, USA). For mVDR, 4.5 ng/well of VDR plasmid was co-transfected with 45 ng/well of the CYP3A4-PXRE-Luc reporter and 20 ng/well of β-galactosidase. For xlVDR and ciVDR/PXR, 67 ng/well of VDR plasmid was co-transfected with 100 ng/well of the reporter tk-UAS-Luc and 20 ng/well of β-galactosidase. For zfVDR and lampVDR, 100 ng/well of VDR plasmid was co-transfected with 150 ng/well tk-UAS-Luc and 20 ng/well of β-galactosidase. For zebrafish PXR, 75 ng/well of VDR plasmid was co-transfected with 100 ng/well tk-UAS-Luc and 20 ng/well of β-galactosidase.

For experiments involving sulfated bile salts or steroids, human SLC21 was co-transfected at 10 ng/well to facilitate compound uptake. On day 3, the cells were washed with Hanks' buffered salt solution (Invitrogen) and then exposed to medium containing the ligands or vehicle to be tested. The medium utilized charcoal-dextran-treated fetal bovine serum (Hyclone, Logan, UT, USA) to reduce background activation. Each drug concentration was performed at least in quadruplicate and repeated in separate experiments for a total of at least three times. For screening experiments, at least three concentrations of each drug were tested. On day 4, the cells were washed with Hanks' buffered salt solution and then exposed to 150 μL lysis buffer (Reporter Lysis Buffer, Promega). Separate aliquots were taken for measurement of β-galactosidase activity (Promega) and luciferase activity (Steady-Glo, Promega).

To facilitate more reliable cross-species comparisons, complete concentration-response curves for ligands were determined in the same microplate as determination of response to a maximal activator. This allows for determination of relative efficacy, ε defined as the maximal response to test ligand divided by maximal response to a reference maximal activator (note than ε can exceed 1). The maximal activators and their concentrations were as follows: hVDR, xlVDR, zfVDR – 1 μM calcitriol (BIOMOL); mVDR and lampVDR – 0.5 μM calcitriol; ciVDR/PXR – 6-formylindolo- [3,2-*b*]-carbazole 20 μM; zebrafish PXR – 20 μM 5α-androstan-3α-ol. All comparisons to maximal activators were done within the same microplate. Luciferase data were normalized to the internal β-galactosidase control and represent means ± SD of the assays.

### Zebrafish primary hepatocyte cultures

Culture of adult zebrafish primary liver cells was adapted from a procedure published by Collodi and colleagues [47]. Zebrafish are sacrificed with MS-222 (Sigma-Aldrich, 0.05% v/v) and immersed in 0.5% bleach diluted in LDF media (50% Leibovitz L-15, 35% Dulbecco's Modified Essential Medium, 15% Ham's F-12, 15 mM HEPES, 0.015% sodium bicarbonate) for 1 min. The livers are microdissected and immersed in 0.5% bleach for 2 min, and then rinsed three times with media. The tissues are then placed in cold LDF media containing 100 units/mL penicillin, 100 μg/mL streptomycin, and 0.25 μg/mL amphotericin. The tissues are minced to 1 mm^3 ^pieces and washed in Hanks' Balanced Salts (HBS) with calcium and magnesium, spun at 500 g for 5 minutes at 4°C. The minceate is digested with 0.25% trypsin for 5 min at room temperature in a 15 mL conical tube. The tube is gently inverted several times to facilitate cell dissociation. The dissociated cells with trypsin are transferred to a tube containing LDF with 5% fetal bovine serum. Fresh trypsin is then added to the remaining liver pieces for 5 min at room temperature and the dissociated cells combined with the other tube of dissociated cells. The total dissociated minceates are spun at 500 g for 5 minutes at 4°C. The supernatant is removed, cells are resuspended in LDF with fetal bovine serum, and spun again at 500 g for 5 minutes at 4°C. The supernatant is again removed and the cells resuspended in 67% LDF containing 5% fetal bovine serum, 100 units/mL penicillin, 100 μg/mL streptomycin, 2 mM glutamine, 50 ng/mL human recombinant growth factor, and 10 μg/mL human insulin. The cells are then plated into 24-well plates (5 × 10^5 ^cells/well) or 96-well plates (9 × 10^4 ^cells/well) plates that have been previously coated with Matrigel diluted 1:47 in LDF (BD Biosciences, San Jose, CA). The cells are cultured at 26°C in ambient atmosphere.

### Zebrafish *in vivo *transcription studies

Adult zebrafish were purchased from a commercial fish supplier and acclimatized for at least two-weeks in 20 L tanks with flow-through freshwater at 22°C. A single dose of 15 mg/kg of 5α-cyprinol, 5β-cyprinol 27-sulfate, 5α-scymnol or 5β-scymnol-27 sulfate, dissolved in saline, was injected intraperitoneally. A control group was injected with saline. After 48 hr, the animals were decapitated and livers of three fish (n = 5 each group) were removed, pooled and weighed, totalizing 15 fish per group. Fish were not fed throughout the experimental period. The numbers of animals used were the minimum necessary to demonstrate consistent effects.

To study the transcription of CYP3C1, ABCB5, PXR, GAPDH and β-actin genes in zebrafish, initial fragments of these genes were identified (Genbank: AW202769, Genbank: BQ284593, Genbank: AAM22215, Genbank: BC083506, Genbank: AF057040, respectively). The PCR primers for CYP3C1 were designed using MacVector Software and primers for MDR1 were designed using Primer 3 Software (Whitehead Institute for Biomedical Research; [75]). Primer pairs for amplifying CYP3C1 fragment were 5'-TTGAGGAGCGGTGGTGAGCATTAG-3' (sense) and 5'-TGGAGAGAGTGAACTTCGGATTCG-3' (antisense) and for amplifying ABCB5 were 5'-CAGAGTGGGCAGACGTACAA-3' (sense) and 5'-TTCGCAGCAGTAAGCAGAAA-3' (antisense). The PCR primers for PXR were 5'-ATGCGGCGACAAATCTACTGGC-3' (sense) and 5'-TGTGAAGTGTGGCAGAGAGGTG-3' (antisense), for amplifying GAPDH were 5'-CCTCCAAGGAGTAGATGTGACC-3' (sense) and 5' GCAGAGGACTTTTATTCCATCG 3' (antisense) and for β-actin were 5'-CGACCCAGACATCAGGGAGTG-3' (sense) and 5'-GTCCAGGGCCACATAGCA-3' (antisense). RNA was isolated using Trizol (Invitrogen) according to manufacturer's instructions. Purity and concentration of RNA of each sample were verified at 260 nm. The RNA integrity was checked by non-denaturing gel electrophoresis using 1 μg of total RNA in each sample.

Two micrograms of total RNA were reversed transcribed using Ominiscript RT Kit (Qiagen, Valencia, CA, USA). cDNA concentration was checked at 260 nm, and the amount adjusted to 1 μg to perform semi-quantitative RT-PCR. The expected size of the PXR, Cyp3C1, abcb5, GAPDH and β-actin fragments were 577, 477, 218, 234 and 550 bp, respectively. Biotools DNA polymerase (Biotools B&M Labs, S.A., Madrid, Spain) kit was used for the PCR reaction using a thermocycler Personal Mastercycler (Eppendörf) with the following PCR program: 5 cycles of 94°C for 5 s, 72°C for 35 s; 5 cycles of 94°C for 5 s, 70°C for 10 s, 72°C for 35 s; and 23, 24, 35, 15 and 24 cycles (PXR, Cyp3C1, abcb5, GAPDH and β-actin, respectively) of 94°C for 5 s, 61, 54, 48, 49 and 61°C for 10 s (PXR, Cyp3C1, abcb5, GAPDH and β-actin, respectively), and 72°C for 35 s. The PXR, CYP3C1, ABCB5 and GAPDH transcript levels were quantified using Gel-Quant™ (Multiplexed Biotechologies) and β-actin was used to normalize the data. The Shapiro-Wilk W test was used to evaluate normality. When data were parametric the analysis was performed using Student t test; otherwise Mann Whitney U test was applied. Differences were considered significant at the 95% confidence level.

### Sea lamprey primary hepatocyte cultures

Culture of sea lamprey primary liver cells was adapted from procedures published by Ma and Collodi [59]. Transformer stage sea lampreys are sacrificed with MS-222 (Sigma-Aldrich, 0.05% v/v) and immersed in 0.5% bleach diluted in LDF media (50% Leibovitz L-15, 35% Dulbecco's Modified Essential Medium, 15% Ham's F-12, 15 mM HEPES, 0.015% sodium bicarbonate) for 1 min. The livers are microdissected and immersed in 0.5% bleach for 2 min, and then rinsed three times with media. The tissues are then placed in cold LDF media containing 100 units/mL penicillin, 100 μg/mL streptomycin, and 0.25 μg/mL amphotericin. The tissues are minced to 1 mm^3 ^pieces and washed in Hanks' Balanced Salts (HBS) with calcium and magnesium, and spun at 500 g for 5 minutes at 4°C. The minceate is digested with 0.25% trypsin for 5 min at room temperature in a 15 mL conical tube. The tube is gently inverted several times to facilitate cell dissociation. The dissociated cells with trypsin are transferred to a tube containing LDF with 10% fetal bovine serum. Fresh trypsin is then added to the remaining liver pieces for 5 min at room temperature and the dissociated cells combined with the other tube of dissociated cells. The total dissociated minceates are spun at 500 g for 5 minutes at 4°C. The supernatant is removed, cells are resuspended in LDF with 10% fetal bovine serum, and spun again at 500 g for 5 minutes at 4°C. The supernatant is again removed and the cells resuspended in 67% LDF containing 5% fetal bovine serum, 100 units/mL penicillin, 100 μg/mL streptomycin, 2 mM glutamine, 50 ng/mL human recombinant growth factor, and 10 μg/mL human insulin. The cells are then plated into 24-well plates (5 × 10^5 ^cells/well) or 96-well plates (9 × 10^4 ^cells/well) plates that have been previously coated with Matrigel diluted 1:47 in LDF (BD Biosciences, San Jose, CA). The cells are cultured at 18°C in ambient atmosphere.

### *Xenopus laevis *primary hepatocyte cultures

*Xenopus laevis *primary hepatocytes were cultured by a protocol adapted from a published report [55]. Frogs are sacrificed with MS-222, rinsed with 70% ethanol, and the liver lobes perfused via the heart, initially with 375 mL Barth (88 mM NaCl, 1 mM K_2_SO_4_, and 10 mM HEPES-NaOH, pH 7.4) containing 0.82 mM MgCl_2 _and 0.1 mg/mL heparin, and then with 25 mL Barth containing 2.22 mM Ca(NO_3_)_2_, 2.74 mM CaCl_2_, and 200 U/mL type I collagenase (Worthington Chemicals, Lakewood, NJ). Livers are minced to fine pieces in 12.5 mL Barth-collagenase solution and then incubated for 10 min at room temperature, periodically disaggregating the liver pieces with a Pasteur pipette. The liver cells are then placed on a shaking incubator for 5 min at room temperature, followed by another 10 min of periodic pipetting to complete disaggregation. Cells are then filtered through a 130 μm Nitex nylon mesh (Fisher Scientific), following by spinning at 500 g for 5 min at 4°C. The supernatant is then aspirated and diluted to 50 mL with Barth plus MgCl_2 _and then allowed to settle while cooled in ice for 30 min. The supernatant is removed, and the cellular pellet is resuspended and subjected to one more round of nylon mesh filtration, dilution in Barth plus MgCl_2_, and settling while cooled in ice. The supernatant is removed and the remaining cells are washed once with Barth plus MgCl_2 _and then centrifuged at 500 g for 5 min at 4°C. The cells are then resuspended in 0.6 × Coon's Modified Ham's F-12 medium (Invitrogen) supplemented with 0.1 × Barth (88 mM NaCl, 1 mM K_2_SO_4_, 10 mM HEPES), 200 U/mL penicillin G, 100 μg/mL streptomycin sulfate, 2 mM glutamine, 0.2% glucose, and 10 μg/mL bovine insulin (Calbiochem), with the final pH adjusted to 7.5. The cells are plated at a density of 2.5–3.0 × 10^4^/mL in 24-well plates that have been previously coated with Matrigel diluted 1:47 in cell culture medium. Cells are cultured at 20°C in ambient atmosphere.

### EROD assay

Cells were incubated with 20 μM 7-ethoxyresorufin (Sigma) in a buffer composed of 50 mM Tris, 0.1 M NaCl, pH 7.8. The production of resorufin was assessed by fluorescence measurement with an excitation wavelength of 530 nm and an emission wavelength of 590 nm, with standards of resorufin made up to determine molar production of resorufin. Reactions were stopped with 1.5 volumes of cold methanol. Measurements were normalized to total protein concentration (BioRad, Hercules, CA, USA).

### HPLC analysis of testosterone metabolites

Testosterone metabolites were identified and quantitated by HPLC. 100 μL of sample, calibration standard, or quality material standard were pipetted into separate microcentrifuge tubes and mixed with 100 μL of methanol. Samples were vortexed and centrifuged at 13,000 rpm for 4 min. The supernatant was then injected into the HPLC.

The samples were separated using an isocratic mobile phase of 60% methanol/40% water on a Lichrospher 100 RP-18 (5 μm, 250 × 4 mm) column (Agilent Technologies, Santa Clara, CA, USA). The flow rate was 1.2 mL/min and total run time was 25 min. The samples were detected by UV absorbance at 242 nm. The retention times for the testosterone metabolites were as follows: 6α-testosterone (4.29 min), 15α-testosterone (4.76 min), 7α-testosterone (5.10 min), 6β-testosterone (5.59 min), 16α-testosterone (6.16 min), 16β-testosterone (8.18 min), 2α-testosterone (8.78 min), and testosterone (18.5 min). Measurements were normalized to total protein concentration (BioRad).

### Flurbiprofen hydroxylation assay

Flurbiprofen hydroxylation activity was measured in intact cultured hepatocytes as an index for CYP2C9 activity. The formation of 4-hydroxyflurbiprofen was measured with reverse-phase HPLC adapted from a previously published method [52]. 100 μL of sample aliquot was diluted with methanol (1:1, v/v) and injected onto a Supelcosil LC-18 column (4.9 × 150 mm, 5 μm) with a mobile phase of 0.02 mol/L potassium phosphate, pH 3.0 buffer/water (60:40) at a flow rate of 1.2 ml/min. Quantification of 4-hydroxyflurbiprofen was done with fluorescence detection (Waters 2475) at 260 nm excitation and 320 nm emission wavelength. Measurements were normalized to total protein concentration (BioRad).

### Phylogenetic analysis

The following sequences were used for phylogenetic analysis (some links are from the Ensembl database [76]): human VDR [Genbank: NM_000376], rhesus monkey VDR [Ensembl: ENSMMUT00000009414], cow VDR [Ensembl: ENSBTAT00000021832], dog VDR [Ensembl: ENCAFT00000014497], mouse VDR [Genbank: NM_009504], chicken VDR [Genbank: AF011356], Japanese quail VDR [Genbank: U12641], *Xenopus laevis *VDR [Genbank: U91849], fugu VDR [Ensembl: NEWSINFRUT00000138841], bastard halibut [Genbank: AB037674], zebrafish VDR [Genbank: AF164512], medaka VDR [Ensembl: ENSORLT00000001311], sea lamprey VDR [Genbank: AY249863], *Ciona intestinalis *VDR/PXR [Genbank: BR000137], human CAR [Genbank: NM_005122], rhesus CAR [Genbank: AY116212], cow CAR [Ensembl: ENSBTAT00000012145], dog CAR [Ensembl: ENSCAFT00000020528], Baikal seal [Genbank: AB109553], mouse CAR [Genbank: NM_009803], pig CAR [Genbank: AB214979], opossum CAR [Ensembl: ENSMODT00000006393], human PXR [Genbank: AF061056], rhesus monkey PXR [Genbank: AF454671], cow PXR [Ensembl: ENSBTAT00000026059], mouse PXR [Genbank: AF031814], rabbit PXR [Genbank: AF188476], opossum PXR [Ensembl: ENSMODT00000023109], chicken PXR [Genbank: AF276753], *Xenopus laevis *PXRα [Genbank: BC041187], *Xenopus tropicalis *PXR [Ensembl: ENSXETT00000039109], fugu PXR [Ensembl: NEWSINFRUT00000171584], medaka PXR [Ensembl: ENSORLT00000022473], *Tetraodon nigriviridis *PXR [Ensembl: GSTENT00026021001], zebrafish PXR [Genbank: AF454674, Genbank: AF502918], ixotid tick (*Amblyomma americanum*) ecdysone receptor [Genbank: AF020187], and purple sea urchin (*Strongylocentrotus purpuratus*) liver × receptor [Genbank: XM_774904]. Sequences were aligned using ClustalW [77] and Tcoffee software [78] and manually adjusted as needed. Phylogeny was inferred using parsimony analysis by PAUP*4.0-beta for UNIX/LINUX (Sinauer Associates, Sunderland, MA, USA) with the ixotid tick ecdysone receptor used as the outgroup. A heuristic search of 100 replicates of random addition plus tree-bisection-reconnection branch swapping was used; to estimate support, 10,000 bootstrap replicates were analyzed. Branch labels indicate bootstrap percentages. The results of the phylogenetic analysis are shown in Figure 4.

## Authors' contributions

EJR performed molecular biology, prepared all the primary cultures of zebrafish, African clawed frog, and sea lamprey cells, and helped draft the manuscript. ACDS and JJM performed the *in vivo *studies in zebrafish. LRH purified bile salts from bile of various animals and provided perspective on evolution of bile salt and steroids. NB provided zebrafish, expert guidance on zebrafish work, and key reagents for the zebrafish cell culture studies. SRM, JO, and RV performed the testosterone 6α-hydroxylation and flurbiprofen hydroxylation assays. MDK conceived of the study, performed the functional assays and phylogenetic analyses, and drafted the manuscript. All authors contributed to, read, and approved the final manuscript.

## Supplementary Material

Additional File 1 Sequence alignment of PXRs and VDRs. Click here for file

Additional File 2 Conservation of VDR and PXR ligand-binding residues across species. Click here for file

Additional File 3 Additional data for *Xenopus laevis*, zebrafish, and sea lamprey VDRs. Click here for file

Additional File 4 Effect of compounds on testosterone hydroxylation in primary cultures of African clawed frog hepatocytes. Click here for file
