# Supplementary material for: Functional evolution of the vitamin D and pregnane X receptors
Source: BMC Evol Biol. 2007 Nov 12;7:222. doi: 10.1186/1471-2148-7-222 (PMC2263054; doi:10.1186/1471-2148-7-222)
Supplement: Additional File 1 — Sequence alignment of PXRs and VDRs. [file 1471-2148-7-222-S1.pdf]

A. DNA-binding domain

```
PXR_human          QICRVCGDKATGYHFNVMTCEGCKGFFRRAMKRNARLRCPFRKGACEITRKTRRQCQACR
PXR_chicken        KVCAVCGDRATGYHFHVMSCEGCKGFFRRSILKGVHFTCPF-TRSCPITKAKRRQCQACR
PXR_fugu           RACGVCGDQAKGYHFNAWTCEGCKGFFRRAIKRTPPLPCQF-LNKCSITKKNRRQCQDCR
VDR_human          RICGVCGDRATGFHFNAMTCEGCKGFFRRSMKRKALFTCPFN-GDCRITKDNRRHCQACR
VDR_mouse          RICGVCGDRATGFHFNAMTCEGCKGFFRRSMKRKALFTCPFN-GDCRITKDNRRHCQACR
VDR_x_laevis       RICGVCGDKATGFHFNAMTCEGCKGFFRRSMKRKAMFTCPFN-GDCRITKDNRRHCQSCR
VDR_zebrafish      PICGVCGDKATGFHFNAMTCEGCKGFFRRSMKRKASFTCPFN-GNCTITKDNRRHCQACR
VDR_lamprey        KVCGVCGDKATGYHFNAMTCEGCKGFFRRSMKRSASFTCPFE-GKCNITKDNRRHCQACR
VDR_PXR_Ciona      ------------MHFGAITCEGCKGFFRRSVKKNASFSCAF-EKKCEINKNNRKHCQACR
CAR_human          RNCVVCGDQATGYHFNALTCEGCKGFFRRTVSKSIGPTCPF-AGSCEVSKTQRRHCPACR


PXR_human          LRKCLESGM
PXR_chicken        LQKCLDVGM
PXR_fugu           LRKCQAIGM
VDR_human          LKRCVDIGM
VDR_mouse          LKRCVDIGM
VDR_x_laevis       LKRCVDIGM
VDR_zebrafish      LKRCIDIGM
VDR_lamprey        LKRCRDIGM
VDR_PXR_Ciona      FNACLAAGM
CAR_human          LQKCLDAGM
```

B. Ligand-binding domain

```
                         Helix-1
PXR_human      141 QGLTEEQRMMIRELMDAQMKTFDTTFSHFKNFRLPGVLSSGCELPESLQAP-SREEA-AK
PXR_chicken        GGLTAEQQELISILIAAHKRTFDSSFSQFQHYQPAVRLC----IPGPCSQS-PPGPG-VP
PXR_fugu           IHLSSQQEETIRELLYGHRKTFDLEFYRFSSFRVRTSTTLFDLSKSLSERL-NIFAV-RG
VDR_human      118 PKLSEEQQRIIAILLDAHHKTYDPTYSDFCQFRPPVRVNDGGGSHPSRPNSRHTPSF-SG
VDR_mouse          PKLSEEQQHIIAILLDAHHKTYDPTYADFRDFRPPIRADVSTGSYSPRP----TLSF-SG
VDR_x_laevis       PKISDEQQKMIDILLEAHRKTFDTTYSDFNKFRPPVRENVDPFRRITRSSSVHTQGSPSE
VDR_zebrafish      PRLSDEQMQIINSLVEAHHKTYDDSYSDFVRFRPPVREGPVTRSASRAASLHSLSDA-SS
VDR_lamprey        PQLLEEQERLIATLIEAHRKTYDASYSDFSQFRPPKRGDGSPECRNATNPFLMSLLN-SD
VDR_PXR_Ciona      TRMTMDEKLLVKTLLKGHRDSYDFAYVEYDTFRGREDGQQEIGNNTENPNG---LDA-AT
CAR_human      103 VQLSKEQEELIRTLLGAHTRHMGTMFEQFVQFRPPAHLFIHHQPL---------------


                                                                   Helix-3
PXR_human      199 WSQVRKDLCSLKVSLQL-RGEDGSVWNYKPP-----ADSGGKEIFSLLPHMADMSTYMFK
PXR_chicken        SASLSPQLDCLDEDVL------------------------PDVFSILPHFADLSTFMIQ
PXR_fugu           SSPSGPASSDV--SSLS-TSARLRGRPETPQTQGGENARRGC-VFTALPHVTDLATCMIH
VDR_human      181 DSSSSCSDHCITSSDM-MDSSSFSNLDLSEEDSDDPSVTLELSQLSMLPHLADLVSYSIQ
VDR_mouse          DSSSNS-DLYTPSLDM-MEPASFSTMDLNEEGSDDPSVTLDLSPLSMLPHLADLVSYSIQ
VDR_x_laevis       DSDVFTSSPDSSEHGFFSASLFGQFEYSSMGGKSGELS--------MLPHIADLVSYSIQ
VDR_zebrafish      DSFNHSPESVDTKLNFSNLLMMYQDSG-SPDSSEEDQQS----RLSMLPHLADLVSYSIQ
VDR_lamprey        MD------------------------ELPKASASGAEAAAGDELSMLPHLADLVSYSIQ
VDR_PXR_Ciona      AVEAQSTTEDSGKQLHL-MLLFQHFLPIYP------FSFDPKA-KQLFQHFCDIMTWGIR
CAR_human      149 -------------------------------------PTLAPVLPLVTHFADINTFMVL


                   Helix-3          Helix-4     Helix-5
PXR_human      253 GIISFAKVISYFRDLPIEDQISLLKGAAFELCQLRFNTVFNAET-GTWECGR--LSY---
PXR_chicken        QVIKFAKEIPAFRGLPIDDQISLLKGATLGICQIQFNTVFNEET-NAWECGQ--HCF---
```

```
PXR_fugu           DIIAFSKSLTDFKSLLIGDQIALLKGATFEVMEIRFNMVFNTKT-GLWECGH--ATY---
VDR_human      240 KVIGFAKMIPGFRDLTSEDQIVLLKSSAIEVIMLRSNESFTMDD-MSWTCGNQDYKY---
VDR_mouse          KVIGFAKMIPGFRDLTSDDQIVLLKSSAIEVIMLRSNQSFTMDD-MSWDCGSQDYKY---
VDR_x_laevis       KIIGFAKMIPGFRDLIAEDQIALLKSSVIEVIMLRSNQSFSLDD-MSWTCGSEDFKYKVD
VDR_zebrafish      KVIGFAKMIPGFRDLTAEDQIALLKSSAIEIIMLRSNQSFSLED-MSWSCGGPDFKYCIN
VDR_lamprey        KVIGFAKMIPGFKELCTEDQISLLKASAIEIIILRSNESFTMED-NSWTCGSNEFKYQIG
VDR_PXR_Ciona      KVIDYCKGIPQFVQLSIVDQIVLLRGGCLEMLVLRSYFAFSCNE-NKYMSDK--FQY---
CAR_human      171 QVIKFTKDLPVFRSLPIEDQISLLKGAAVEICHIVLNTTFCLQT-QNFLCGP--LRY---

                                 Helix-7             Helix-8           Helix-9
PXR_human      308 CLEDT-AGGF----QQLLLEPMLKFHYMLKKLQLHEEEYVLMQAISLFSPDRPGVLQHRV
PXR_chicken        TIKDGALAGF----QQIYLEPLLKFHISLKKLRLHEAEYVLLVAMLLFSPDHASVTQRDF
PXR_fugu           CIEDAVRAGF----QPLFLEPLLKFHHTLRNLGLEEEEYVLMQALSLFSPDRPGVQQHSV
VDR_human      297 RVSDVTKAGH----SLELIEPLIKFQVGLKKLNLHEEEHVLLMAICIVSPDRPGVQDAAL
VDR_mouse          DITDVSRAGH----TLELIEPLIKFQVGLKKLNLHEEEHVLLMAICIVSPDRPGVQDAKL
VDR_x_laevis       D---VTQAGH----NMELLEPLVKFQVGLKKLDLHEEEHVLLMAICILSPDRPGLQDKAL
VDR_zebrafish      D---VTKAGH----TLELLEPLVKFQVGLKKLKLHEEEHVLLMAICLLSPDRPGVQDHVR
VDR_lamprey        D---VMQAGH----KLELLEPLVKFQVNMKKLDLHEAEHVLLMAICLFSPDRPGVQDRCR
VDR_PXR_Ciona      KPSDFLQAGG----NKEFVEKYNSLHIRMRKMKLQVEEICLLLALVLFSPDRPGLEDQAK
CAR_human      226 TIEDGARVGF----QVEFLELLFHFHGTLRKLQLQEPEYVLLAAMALFSPDRPGVTQRDE

                      Helix-9                  Helix-10
PXR_human      363 IDQLQEQFAITLKSYIECNR-PQPAHRFLFLKIMAMLTELRSINAQHTQRLLRIQDIHPFA
PXR_chicken        VDQLQEKVALTLKSYIDHRH-PMPEGRFLYAKLLLLLTELQTLKMENTRQILHIQDLSSM-
PXR_fugu           IDKIHENLALALKTRIELKR-TGPEKHMLYPKVLSCLTEMRTMNEEYSKQVLQIQDIQPNV
VDR_human      353 IEAIQDRLSNTLQTYIRCRH-PPPGSHLLYAKMIQKLADLRSLNEEHSKQYRCLSFQPECS
VDR_mouse          VEAIQDRLSNTLQTYIRCRH-PPPGSHQLYAKMIQKLADLRSLNEEHSKQYRSLSFQPENS
VDR_x_laevis       VESIQDRLSSTLQTYILCKH-PPPGSRLLYAKMIQKLADLRSLNEEHSKQYRSISFLPEHS
VDR_zebrafish      IEALQDRLCDVLQAYIRIQH-P--GGRLLYAKMIQKLADLRSLNEEHSKQYRSLSFQPEHS
VDR_lamprey        IEEVQEHLTETLRAYIACRH-PLSCKHMLYTKMVEKLTELRSLNEEHSKQYLQISQDAVNK
VDR_PXR_Ciona      VEQMQDCVANTLQAYEYTHK-PPNESSFLQARTMYCLPILRTINMLFAQNIMSLQTNEKDM
CAR_human      282 VDQLQEEMALTLQSYIKGQQ-RRPRDRFLYAKLLGLLAELRSINEAYGYQIQHIQGLSAMM

PXR_human      422 ATPLMQELFGITGS
PXR_chicken        -TPLLSEIIS
PXR_fugu           VIPPLLMEMVS
VDR_human      412 SMKLTPLVLEVFGNEIS
VDR_mouse          SMKLTPLVLEVFGNEIS
VDR_x_laevis       SMKLTPLMLEVFSDEIP
VDR_zebrafish      SMQLTPLVLEVFGSEVS
VDR_lamprey        KEDLPPLLLEVFGNPTA
VDR_PXR_Ciona      N-PLILEVNNSADDED
CAR_human      341 MPLLQEICS
```

Additional file 1: Sequence alignment of (A) DNA-binding domain and (B) ligand-binding domain of three PXRs, five VDRs, *Ciona intestinalis* VDR/PXR, and human CAR. The ligand-binding domain is annotated with the α-helices [1]. Accession numbers are: human PXR [Genbank:AF061056], chicken PXR [Genbank:AF276753], takifugu PXR [Ensembl,http://www.ensembl.org:NEWSINFRUT00000171584], human VDR [Genbank:NM_00376], mouse VDR [Genbank:NM_008504], *Xenopus laevis* VDR [Genbank:U91849], zebrafish VDR [Genbank:AF164512], sea lamprey VDR [Genbank:AY249863], *Ciona intestinalis* VDR/PXR [Genbank:BR000137], and human

CAR [Genbank:NM_005122]. Note that some N-terminal sequence is unavailable for the *Ciona* VDR/PXR.

Reference

1. Moore LB, Maglich JM, McKee DD, Wisely B, Willson TM, Kliewer SA, Lambert MH, Moore JT: **Pregnane X receptor (PXR), constitutive androstane receptor (CAR), and benzoate X receptor (BXR) define three pharmacologically distinct classes of nuclear receptors**. *Mol Endocrinol* 2002, **16:**977-986.
