# Supplementary material for: Functional evolution of the vitamin D and pregnane X receptors
Source: BMC Evol Biol. 2007 Nov 12;7:222. doi: 10.1186/1471-2148-7-222 (PMC2263054; doi:10.1186/1471-2148-7-222)
Supplement: Additional File 2 — Conservation of VDR and PXR ligand-binding residues across species. [file 1471-2148-7-222-S2.pdf]

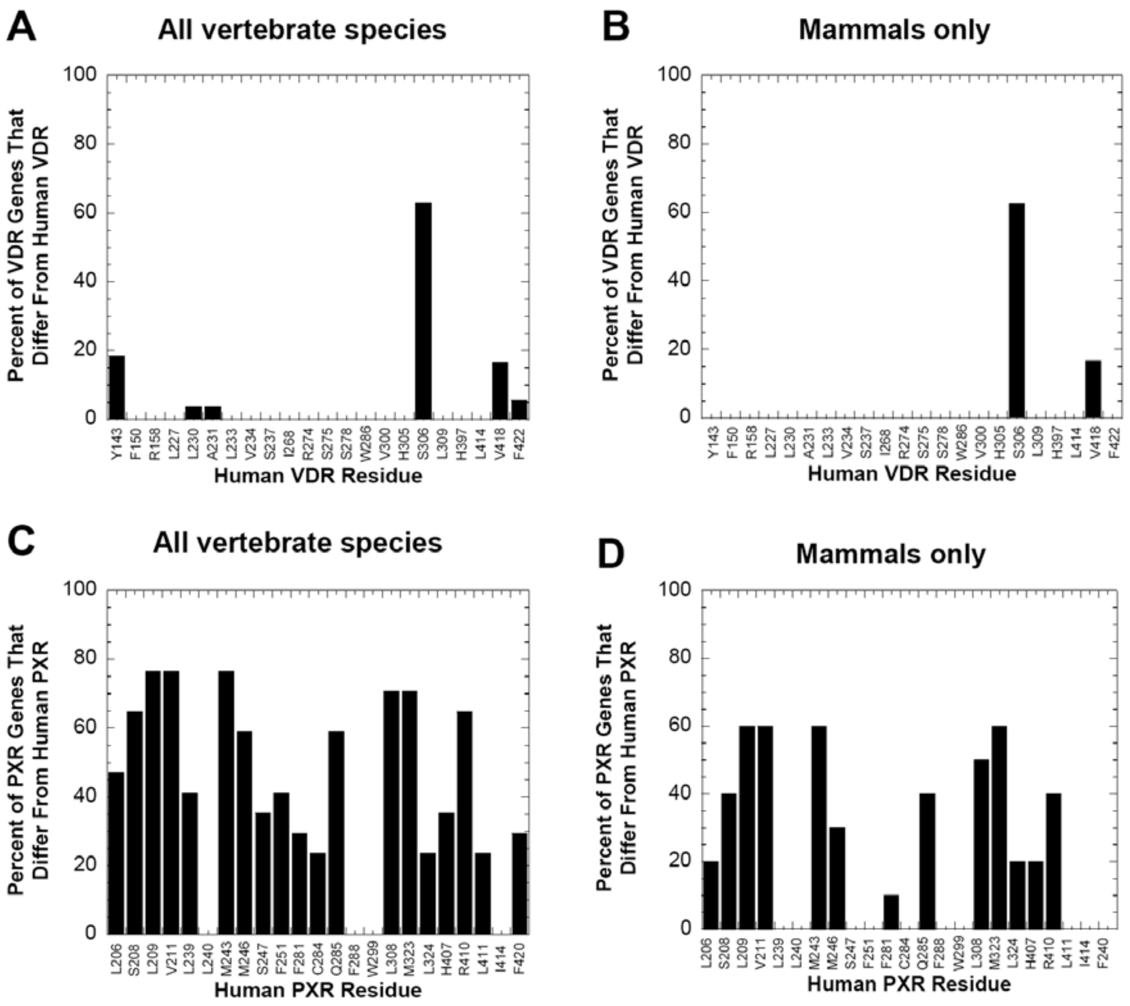

Additional file 2 – Conservation of VDR and PXR ligand-binding residues across species. The ligand-binding residues in VDRs are from x-ray crystallographic structures of human [1-3], rat [4, 5], and zebrafish VDRs [6, 7]. (**A**) Of the 22 amino acids residues shown to interact with ligands at human, rat, and/or zebrafish VDRs, only 6 residues show any sequence variation across vertebrate species (including the sea lamprey). (**B**) Only 2 of 22 ligand-binding residues show any variation across mammalian VDRs. (**C**) In contrast to VDRs, PXR ligand-binding residues, as determined from multiple crystal structures of human PXR [8-12], are quite variable across species. Only 4 of 23 positions are *conserved* throughout the vertebrate PXRs, with a number of the positions showing extensive sequence variation across species. (**D**) Even within mammalian species, only 10 of 23 ligand-binding residues are conserved. VDR analysis was based on 27 total species (8 mammals) (Ensembl sequences can be found at http://www.ensembl.org): human [GenBank:NM_00376], chimpanzee [Ensembl:ENSPTRT00000009010], cotton-top tamarin monkey [Genbank:AF354232], rhesus monkey [Ensembl:ENSMMUT00000009414], cow [Ensembl:ENSBTAT00000021832], dog [Ensembl:ENCAFT00000014497], mouse [GenBank:NM_008504], rat [Genbank:NM_017058], chicken

[GenBank:AF011356], Japanese quail [GenBank:U12641], *Xenopus laevis* [GenBank:U91849], *Xenopus tropicalis* [Ensembl:ENSXETT00000023339], Nile crocodile [Genbank:AJ011391], snake (*Elaphe* sp.) [Genbank:AJ286866], red-eared slider turtle [Genbank:AJ286870], giant toad (*Bufo marinus*) [Genbank:AY268062], tokay (*Gekko gecko*) [Genbank:AY254096], Asiatic carp [Genbank:AJ784084], fugu VDRα [Ensembl:SINFRUT00000174563], fugu VDRα [Ensembl:SINFRUT00000165525], Japanese flounder VDRα [GenBank:AB037674], Japanese flounder VDRβ [GenBank:AB037673], zebrafish [GenBank:AF164512], medaka [Ensembl:ENSORLT00000001311], salmon [Genbank"AJ780914], sea bass [Genbank:AM040727], and sea lamprey [GenBank:AY249863]. Reliable sequence was not available for *Xenopus tropicalis* for R158; sequences were not available for horse, crocodile, gecko, toad, snake, turtle, carp, and salmon for H397; sequences were not available for dog, horse, crocodile, gecko, toad, snake, turtle, carp, and salmon for L414, V418, and F422. PXR data was based on analysis of 17 total species (10 mammalian species): human [GenBank:AF061056], chimpanzee [Ensembl:ENPTRT00000028510], rhesus monkey [GenBank:AF454671], cow [Ensembl:ENSBTAT00000026059], dog [Genbank:AF454670], pig [Genbank:AF454672], mouse [AF031814], rat [Genbank:AF151377], rabbit [GenBank:AF188476], opossum [Ensembl:ENSMODT00000023109], chicken [GenBank:AF276753], *Xenopus laevis* BXRα [GenBank:BC041187], *Xenopus tropicalis* [Ensembl:ENSXETT00000039109], fugu [Ensembl:NEWSINFRUT00000171584], medaka [Ensembl:ENSORLT00000022473], *Tetraodon nigriviridis* [Ensembl:GSTENT00026021001], and zebrafish [GenBank:AF454674, GenBank:AF502918].

References

1. Tocchini-Valentini G, Rochel N, Wurtz JM, Mitschler A, Moras D: **Crystal structures of the vitamin D receptor complexed to superagonist 20-epi ligands**. *Proc Natl Acad Sci U S A* 2001, **98:**5491-5496.
2. Tocchini-Valentini G, Rochel N, Wurtz J-M, Moras D: **Crystal structure of the vitamin D receptor liganded with the vitamin D side chain analogues calicotriol and seocalcitol, receptor agonists of clinical importance. Insights into a structural basis for the switching of calcipotriol to a receptor antagonist by a further side chain modification**. *J Med Chem* 2004, **47:**1956-1961.
3. Rochel N, Wurtz JM, Mitschler A, Klaholz B, Moras D: **The crystal structure of the nuclear receptor for vitamin D bound to its natural ligand**. *Mol Cell* 2000, **5:**173-179.
4. Vanhooke JL, Benning MM, Bauer CB, Pike JW, DeLuca HF: **Molecular structure of the rat vitamin D receptor ligand binding domain complexed with 2-carbon-substituted vitamin D_3 hormone analogues**

and a LXXLL-containing coactivator peptide. *Biochemistry* 2004, **43:**4101-4110.
5. Vanhooke JL, Tadi BP, Benning MM, Plum LA, DeLuca HF: **New analogs of 2-methylene-19-nor-(*20S*)-1,25-dihydroxyvitamin D_3 with conformationally restricted side chains: evaluation of biological activity and structural determination of VDR-bound conformations**. *Arch Biochem Biophys* 2007, **460:**161-165.
6. Ciesielski F, Rochel N, Mitschler A, Kouzmenko A, Moras D: **Structural investigation of the ligand binding domain of the zebrafish VDR in complexes with 1α,25$(OH)_2D_3$ and Gemini: purification, crystallization and preliminary X-ray diffraction analysis**. *J Steroid Biochem Mol Biol* 2004, **89-90:**55-59.
7. Ciesielski F, Rochel N, Moras D: **Adaptability of the Vitamin D nuclear receptor to the synthetic ligand Gemini: remodelling the LBP with one side chain rotation**. *J Steroid Biochem Mol Biol* 2007, **103:**235-242.
8. Chrencik JE, Orans J, Moore LB, Xue Y, Peng L, Collins JL, Wisely GB, Lambert MH, Kliewer SA, Redinbo MR: **Structural disorder in the complex of human PXR and the macrolide antibiotic rifampicin**. *Mol Endocrinol* 2005, **19:**1125-1134.
9. Watkins RE, Wisely GB, Moore LB, Collins JL, Lambert MH, Williams SP, Willson TM, Kliewer SA, Redinbo MR: **The human nuclear xenobiotic receptor PXR: structural determinants of directed promiscuity**. *Science* 2001, **292:**2329-2333.
10. Watkins RE, Maglich JM, Moore LB, Wisely GB, Noble SM, Davis-Searles PR, Lambert MH, Kliewer SA, Redinbo MR: **2.1 Å crystal structure of human PXR in complex with the St. John's wort compound hyperforin**. *Biochemistry* 2003, **42:**1430-1438.
11. Xue Y, Chao E, Zuercher WJ, Willson TM, Collins JL, Redinbo MR: **Crystal structure of PXR-T1317 complex provides a scaffold to examine the potential for receptor antagonism**. *Bioorg Med Chem* 2007, **15:**2156-2166.
12. Xue Y, Moore LB, Orans J, Peng L, Bencharit S, Kliewer SA, Redinbo MR: **Crystal structure of PXR-estradiol complex provides insights into endobiotic recognition**. *Mol Endocrinol* 2007, **21:**1028-1038.
