# Supplementary material for: Functional evolution of the vitamin D and pregnane X receptors
Source: BMC Evol Biol. 2007 Nov 12;7:222. doi: 10.1186/1471-2148-7-222 (PMC2263054; doi:10.1186/1471-2148-7-222)
Supplement: Additional File 4 — Effect of compounds on testosterone hydroxylation in primary cultures of African clawed frog hepatocytes. [file 1471-2148-7-222-S4.pdf]

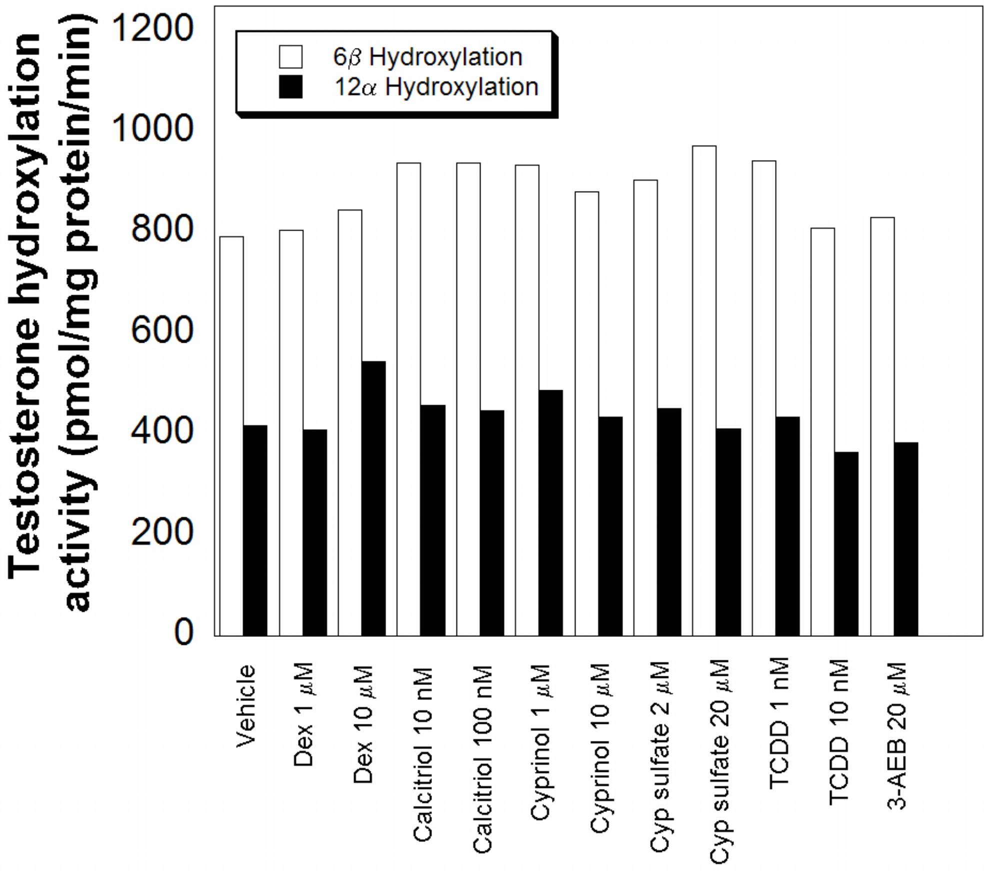

Additional file 4: Effect of compounds on testosterone hydroxylation in primary cultures of African clawed frog hepatocytes. Both 6β- and 12α-hydroxylation of testosterone are evident in the frog hepatocytes. Neither activity is induced by dexamethasone (Dex), calcitriol, 5α-cyprinol, 5α-cyprinol 27-sulfate (Cyp sulfate), TCDD, or 3-aminoethylbenzoate (3-AEB).
